# New Sources, Differentiation, and Therapeutic Uses of Mesenchymal Stem Cells 2.0

**DOI:** 10.3390/ijms24043938

**Published:** 2023-02-15

**Authors:** Sung-Chul Jung, Saeyoung Park

**Affiliations:** 1 Department of Biochemistry, College of Medicine, Ewha Womans University, Seoul 07804, Republic of Korea; 2 Graduate Program in System Health Science and Engineering, Ewha Womans University, Seoul 07804, Republic of Korea

For the clinical application of mesenchymal stem cells (MSCs), the optimization of biological products (e.g., MSCs, extracellular vesicles, or spheroids) has to be performed based on their molecular properties and mechanisms of action. The paracrine function of MSCs via the secretome, and the unique stem cell properties of MSCs, such as proangiogenic, anti-inflammatory, immunomodulatory, and antioxidative stress activities, represent the mechanisms of action underlying the use of MSCs for therapeutic purposes [1,2]. As multipotent cells, MSCs definitively have the capacity to differentiate into mesodermal lineages, including chondroblasts, osteoblasts, and adipocytes. The bone marrow, adipose tissues, Wharton’s jelly, umbilical cord blood, amniotic fluid, placenta, and tonsils have been studied as sources of MSCs, and MSCs derived from these tissues have been shown to be suitable as cell therapy products [3,4].

The use of MSCs constitutes a promising therapeutic approach, as their beneficial effects in various disease conditions have been demonstrated; however, the progress in the clinical application and therapeutic use of MSCs is limited and further behind than expected. Moreover, many obstacles remain to be addressed before MSCs can be applied in the real world. For example, the minimization of donor-dependent and bioprocess variabilities is required for the large-scale expansion of MSCs for use as allogeneic therapies [5]. In the case of cell therapy using differentiated cells, the optimization of the quality of the cell therapy candidates is very difficult. In addition to the discovery of novel tissue sources of MSCs, the differentiation potential of MSCs derived from various sources, the optimization of the quality of MSCs (including their differentiated cell products for therapeutics), and the accurate molecular mechanisms underlying the action of cell therapy products for disease conditions warrant further study. This Special Issue, which is entitled “New Sources, Differentiation, and Therapeutic Uses of Mesenchymal Stem Cells 2.0”, includes eight articles, one of which is a review that discusses the connection between MSC therapy and osteoclasts in osteoarthritis (OA) [6].

Tonsil-derived mesenchymal stem cells (T-MSCs) can be differentiated into endoderm lineages, especially parathyroid-hormone (PTH)-releasing cells [4,7]. Kim et al. performed quality optimization by standardizing the differentiation rate for a better clinical application of human T-MSCs differentiated into PTH-releasing cells to overcome the donor-dependent variation of T-MSCs [8]. A standardized efficiency of differentiation into PTH-releasing cells was achieved by initiating differentiation at a high cell density [9,10,11]. This finding provides a potential solution for overcoming the limitations caused by donor-dependent variations via the establishment of a standardized differentiation protocol for the clinical application of MSC therapy using differentiated cells. The osteogenic differentiation of MSCs is a standard procedure in bone tissue engineering. As a promising field for clinical applications, many cell culture media exist that promote osteogenic differentiation. Glossner et al. explored the effects of different basal cell culture media on the osteogenic differentiation of MSCs, as evaluated using radioactive ^99m^Tc-HDP labeling and quantitative alizarin red staining [12]. These authors reported that all media, with the exception of “Bernese medium”, were suitable for osteogenic differentiation, whereas there was evidence that DMEM low glucose (DMEM LG) is partly superior when used for the expansion and differentiation of BM-hMSCs. This study suggests that the choice of the cell culture medium can result in significant differences in the osteogenic cellular process [12]. Ramalingam et al. reported the therapeutic role of a conditioned medium derived from neural-induced adipose tissue-derived MSCs (NI-hADSC-CMs) against rotenone-induced Parkinson’s disease-like impairments, and the neuroprotective effects of NI-hADSC-CMs on the autophagy signaling pathways [13].

The preconditioning of MSCs, including exposure to hypoxia, growth factors, hormones, and pharmacological or chemical agents, has been shown to optimize the paracrine potency and therapeutic efficacy of MSCs [14,15,16]. Jung et al. reported that thrombin preconditioning of human Wharton’s jelly-derived MSCs improved their therapeutic potential and attenuated brain injury, including progressive ventricular dilatation, gliosis, cell death, inflammation, and neurobehavioral functional impairment, more effectively in newborn rats [16]. TGFβ-treated placenta-derived MSCs (PMSCs) have an anti-adipogenic effect in thyroid-associated ophthalmopathy (TAO) [17]. Shin et al. reported that TGFβ-hPMSCs exhibited anti-inflammatory and anti-fibrotic functions, and suggested that TGFβ-hPMSCs may represent a new and safe method that can be used to promote the anti-adipogenic function of hPMSCs to treat patients with TAO [17].

A significant lack of donor organs might restrict the opportunity to obtain tissue-specific scaffolds for tissue engineering technologies. The development of decellularization protocols for human donor organs that are unsuitable for transplantation has been suggested as a solution to this problem. Sevastianov et al. reported the development of a protocol for obtaining a biocompatible tissue-specific scaffold from decellularized fragments with pronounced signs of human pancreas lipomatosis with preserved basic fibrillary proteins of a pancreatic tissue extracellular matrix [18]. The scaffold supports the adhesion and proliferation of human adipose-derived stem cells (hADSCs) and prolongs the viability and insulin-producing function of pancreatic islets [18]. Tissues from postmortem donors were also proposed as valuable alternative sources for the isolation of primary cells with MSC-like properties [19,20]. Haring et al. reported the identification of an optimal tissue source among three knee and peri-knee tissues for the isolation of primary cells with MSC-like properties, and described the effect of the postmortem time on the properties of these cells [21]. In fact, the success rate of primary cell isolation depends on the postmortem time. Synovium and periosteum cells isolated at more than 48 h postmortem exhibited improved osteogenic and chondrogenic potential [21]. This study suggests that knee and peri-knee tissues from donors, even at three days postmortem, may be suitable strategic sources of MSCs for regenerative procedures [21].

Ibáñez et al. reviewed the need for further studies that can support MSCs as a therapeutic tool for osteoclasts and their consequences on the osteoarthritic joint [6]. Most research aimed at the development of treatments for OA is focused on chondrocytes and cartilage improvement; however, there are few studies referring to the therapeutic effects of MSCs on osteoclasts (OCLs). The improvement of OA through MSC therapy may be related not only to its chondroprotective effect, but also to its blocking activity on OCLs [22,23]. However, the opposite results reported in other studies demonstrating the osteoclastogenic activity of MSCs suggest that more complex interactions may exist between MSCs and osteoclasts [24,25].

The optimization of MSC production, the enhancement of the efficacy of MSCs in clinical practice, the identification of novel sources for regenerative medicine (such as postmortem tissues and decellularized organ fragments), and the importance of the interaction between MSCs and the host environment have been presented in this issue. Together with the previous issue [1], continuous and accumulated efforts will eventually yield successful results regarding the clinical use of MSCs and their products in clinical practice.
